# Spontaneous Renal Hemorrhage in a Diabetic Male Patient With Benign Prostatic Hyperplasia: A Case Report

**DOI:** 10.7759/cureus.88139

**Published:** 2025-07-17

**Authors:** Sergio Flores González, Jose Maria Zepeda Torres, Carolina Topete Rodríguez, Jose Martinez Chavez, Cesar Emmanuel Vazquez Padilla, Andrea Michelle Salas Carlock, Martha Berenice Ochoa Mariscal

**Affiliations:** 1 Surgery, Hospital General de Zona 1, Instituto Mexicano del Seguro Social, Tepic, MEX; 2 Surgery, Centro Médico Nacional de Occidente, Instituto Mexicano del Seguro Social, Guadalajara, MEX; 3 Surgery, Universidad de Guadalajara, Guadalajara, MEX; 4 Surgery, Hospital General de Zona No. 89, Instituto Mexicano del Seguro Social, Guadalajara, MEX; 5 General Surgery, Hospital de Especialidades, Centro Médico Nacional de Occidente, Instituto Mexicano del Seguro Social, Guadalajara, MEX

**Keywords:** explorative laparotomy, open nephrectomy, spontaneous renal hemorrhage, wunderlich's syndrome, wunderlich syndrome

## Abstract

Wunderlich syndrome, defined as spontaneous nontraumatic renal hemorrhage, is a rare clinical entity. We present a case of Wunderlich syndrome in a 62-year-old male patient with type 2 diabetes mellitus and benign prostatic hyperplasia (BPH) who presented with recurrent hematuria and was found to have a Grade III left renal hematoma. The patient underwent exploratory laparotomy and left nephrectomy. The postoperative course was marked by hematuria, necessitating close monitoring and management. Nephrology consultation was obtained, with recommendations for avoiding nephrotoxic agents and careful monitoring of renal function. The patient was eventually discharged without complications. This case highlights the importance of considering spontaneous renal hemorrhage in patients with diabetes and BPH, as well as the potential for successful surgical management.

## Introduction

Wunderlich syndrome is a rare clinical entity, with its true incidence not well established due to its infrequent and often underreported presentation. The syndrome is characterized by spontaneous, nontraumatic renal hemorrhage, most commonly presenting in adults, with a slight female predominance attributed to the higher incidence of angiomyolipoma in women. The majority of cases are associated with underlying renal neoplasms; angiomyolipoma and renal cell carcinoma together account for approximately 60-65% of cases, while renal vascular diseases (including aneurysms, arteriovenous malformations, and vasculitis) contribute to 20-30% of cases. Less common etiologies include renal cystic disease, infections, calculi, and coagulopathies. Idiopathic cases, in which no underlying cause is identified, are also reported but are less frequent [1,2].

The syndrome can occur across a wide age range, but older age is associated with a higher likelihood of renal cell carcinoma as the underlying etiology. In a series of 28 events in 26 patients, the mean age was not specified; however, the data support an association between advanced age and malignant causes [2]. There is no evidence to suggest a significant geographic or racial predilection. The rarity of Wunderlich syndrome and its variable presentation make large-scale epidemiologic data limited, and most knowledge is derived from case series and institutional reviews [1,2]. Given the unusual combination of risk factors and the successful management, we present an interesting case of Wunderlich syndrome in a patient with diabetes and benign prostatic hyperplasia (BPH), highlighting the diagnostic and therapeutic challenges in this setting.

## Case presentation

**Figure 1:**
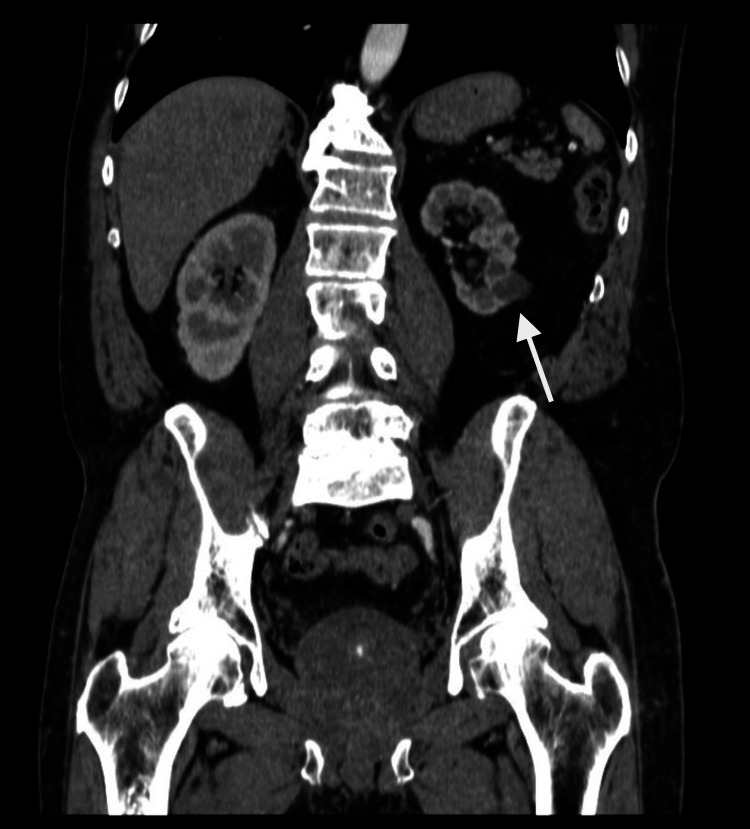
Previous CT scan showing a renal hematoma measuring 1.5 x 1 cm in largest diameter (arrow), located in the lower pole

**Figure 2:**
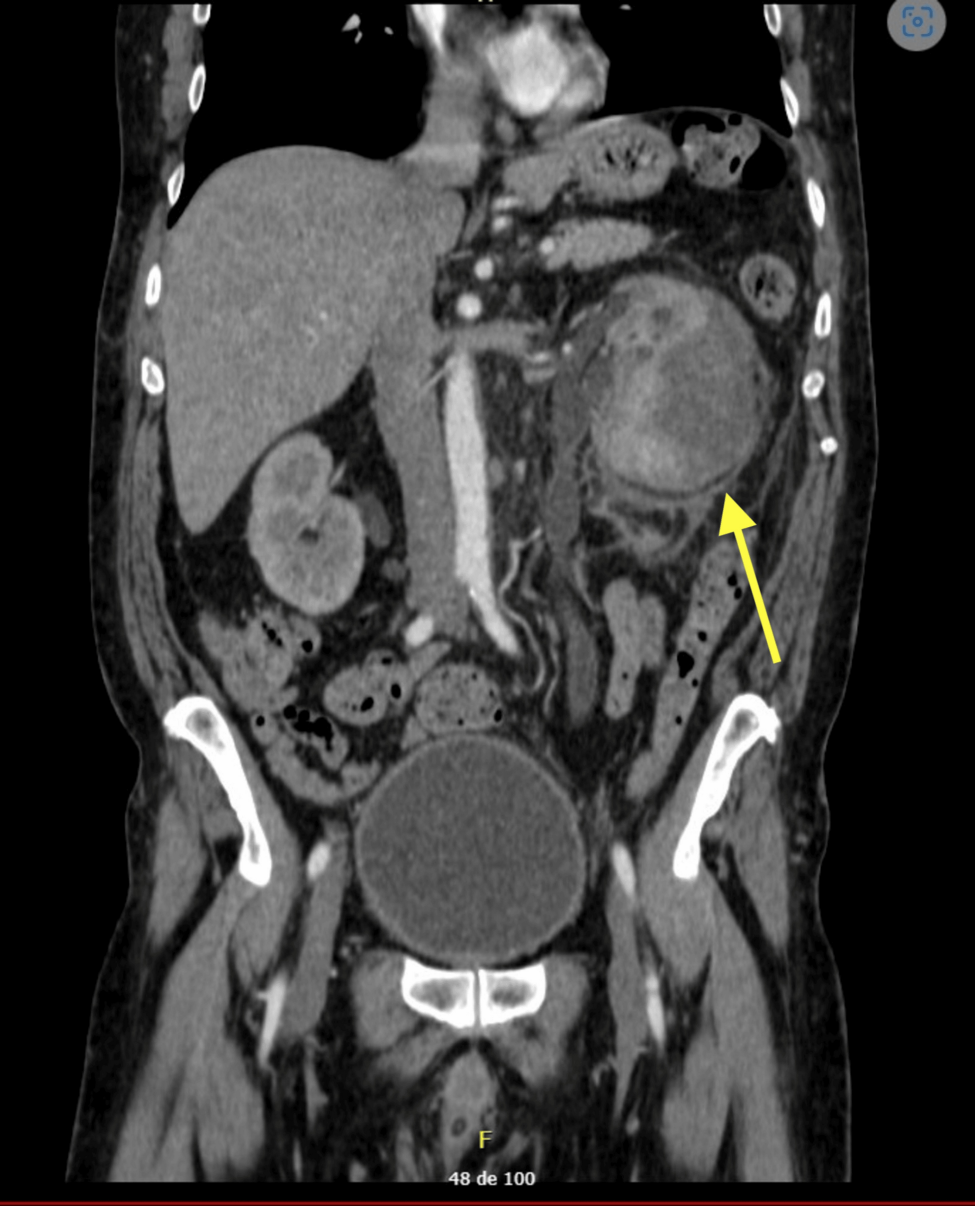
Axial CT showing a renal hematoma observed in the coronal section (yellow arrow) that displaces the renal parenchyma and causes hydronephrosis

A 62-year-old male with a notable history of type 2 diabetes mellitus and BPH presented with recurrent hematuria, leading to the discovery of a Grade III left renal hematoma via CT urography imaging (Figures 1, 2).

**Figure 3:**
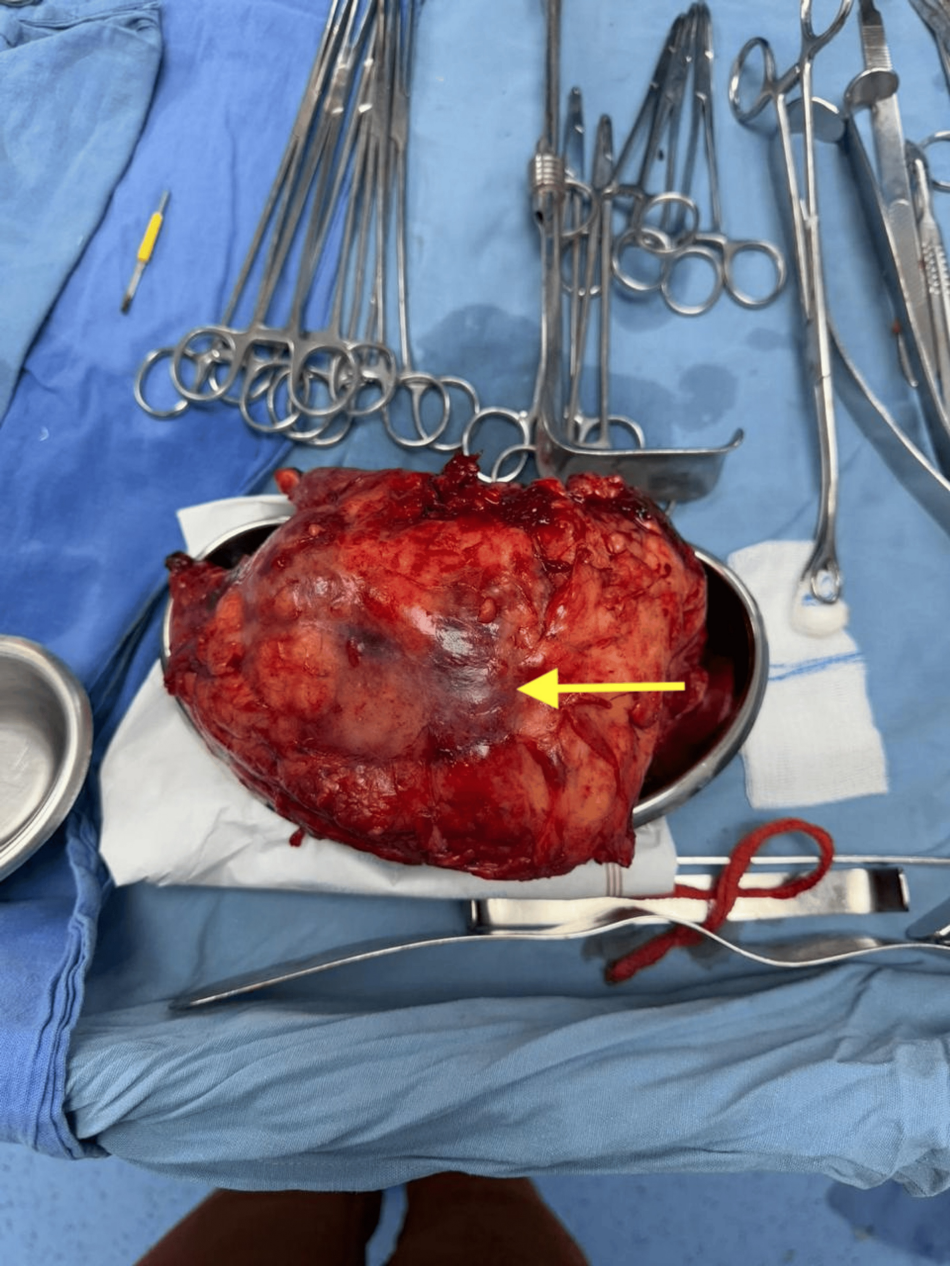
Product of nephrectomy. The arrow indicates a renal hematoma at the lower pole

The patient underwent an exploratory laparotomy and left nephrectomy. Intraoperative findings revealed a severely compromised left kidney, characterized by a thickened Gerota's fascia heavily infiltrated by hematoma and firmly adhered to the adjacent colon and psoas muscle (Figure 3).

Additionally, a large hematoma was observed, significantly displacing the renal unit. Postoperatively, the patient's course was marked by hematuria and fluctuations in urine output, which necessitated close monitoring and management. Given the patient's complex medical history, including a 30-year history of type 2 diabetes, BPH, and a significant smoking history, a nephrology consultation was obtained. The nephrology team recommended avoiding nephrotoxic agents, carefully adjusting medications based on the patient's glomerular filtration rate, and implementing a plan for annual monitoring involving body mass index, urinalysis, and serum creatinine assessments. Importantly, the patient was eventually discharged without complications.

## Discussion

Wunderlich syndrome is defined as the spontaneous, nontraumatic hemorrhage into the subcapsular and perirenal spaces of the kidney, most often presenting acutely. The classic clinical presentation is described by Lenk's triad: sudden onset of flank pain, a palpable flank mass, and hypovolemic shock, although not all patients exhibit the full triad, and symptoms can be nonspecific [3]. The most common etiologies are renal neoplasms, with angiomyolipoma being the most frequent benign cause and renal cell carcinoma the most common malignant cause. Other causes include vascular abnormalities (such as polyarteritis nodosa, renal artery aneurysms, and arteriovenous malformations), cystic renal diseases, infections, nephrolithiasis, nephritis, and coagulation disorders [3-5].

Diagnosis relies on cross-sectional imaging, particularly CT, which is essential for identifying the hemorrhage, assessing its extent, and determining the underlying etiology [3,5]. Management depends on hemodynamic stability and the underlying cause, ranging from conservative measures and selective embolization to surgical intervention in cases of ongoing bleeding or hemodynamic compromise [4,5].

Surgical management options for Wunderlich syndrome depend on the underlying etiology, hemodynamic stability, and extent of renal involvement. In cases of spontaneous renal hemorrhage due to neoplasms such as angiomyolipoma or renal cell carcinoma, surgical interventions include partial nephrectomy or radical nephrectomy, particularly when there is ongoing hemorrhage, suspicion of malignancy, or failure of less invasive measures. In hemodynamically unstable patients or when the renal parenchyma is extensively destroyed, emergency nephrectomy may be required. For select cases, especially when the bleeding source is localized and renal preservation is feasible, partial nephrectomy or hematoma evacuation may be considered [1,2].

If Wunderlich syndrome is left untreated, the most significant and immediate complication is life-threatening hemorrhagic shock due to ongoing retroperitoneal bleeding, which carries a high risk of mortality [6-8]. Additional complications include the development of large perinephric or subcapsular hematomas, which can lead to compression of the renal parenchyma and subsequent loss of renal function. Persistent or recurrent bleeding may also result in the formation of vascular complications such as pseudoaneurysms, as described in cases where subcapsular hematomas strip the renal capsule and tear cortical arteries [4]. Furthermore, ongoing hemorrhage and hematoma formation can predispose to secondary infection, abscess formation, and, in rare cases, abdominal compartment syndrome. The cumulative effect of these complications is a substantial risk of irreversible renal damage and multi-organ failure if prompt intervention is not undertaken [1-4].

## Conclusions

This case is reported to underscore several important lessons in the management of Wunderlich syndrome. It highlights the necessity of maintaining a high index of suspicion for spontaneous renal hemorrhage in patients presenting with hematuria, especially in those with complex comorbidities such as diabetes and BPH. Despite the patient's age and clinical background suggesting potential malignancy, a thorough diagnostic evaluation was crucial to exclude less common causes, emphasizing the importance of careful assessment in such cases. The successful outcome following surgical intervention demonstrates that, with appropriate patient selection and vigilant management, prompt and decisive treatment can lead to positive results, even in complex scenarios. Additionally, this case underscores the importance of long-term follow-up, focusing on renal function, blood pressure control, and recurrence prevention. Ultimately, reporting this case aims to contribute to the understanding of Wunderlich syndrome in older patients with metabolic and urological comorbidities and to stimulate further research into the potential links between conditions like diabetes, prostatic disease, and spontaneous renal hemorrhage.
